# Model-Based Magnetization Transfer Imaging Markers to Characterize Patients and Asymptomatic Gene Carriers in Huntington’s Disease

**DOI:** 10.3389/fneur.2017.00465

**Published:** 2017-09-06

**Authors:** Roland Wiest, Jean-Marc Burgunder, Claus Kiefer

**Affiliations:** ^1^Support Center of Advanced Neuroimaging (SCAN), Institute for Diagnostic and Interventional Neuroradiology, University of Bern, Inselspital, University Hospital, Bern, Switzerland; ^2^Neurology, University of Bern, Inselspital, University Hospital, Bern, Switzerland; ^3^Sichuan University, Xiangya Hospital, Central South University, Changsha, China; ^4^Sun Yat Sen University, Chengdu, China

**Keywords:** magnetization transfer imaging, Huntington’s disease, caudate nucleus, classification, pre-Huntington’s disease gene carriers

## Abstract

**Background and purpose:**

Huntington’s disease (HD) is a chronic progressive neurodegenerative disorder with a long presymptomatic period that opens a window for potential therapies aimed at neuroprotection. Neuroimaging offers the potential to monitor disease-related progression of the disease burden (DB) using model-based magnetization transfer imaging.

**Materials and methods:**

We have conducted a cross-sectional study to stratify healthy age-matched controls, premanifest and symptomatic HD patients (*n* = 30) according to their macromolecular depositions in the caudate nucleus. We employed a binary spin-bath magnetization transfer (MT) method for a quantitative description of macromolecule deposits and interactions with their adjacent environment.

**Results:**

A region-of-interest based fuzzy clustering analysis identified representative clusters for several stages of the disease course related to its progression: one cluster represented subjects with a high DB <268 that encompassed all symptomatic HD patients and one presymptomatic gene carrier. The next cluster represented the presymptomatic gene carriers with a very low DB >230 and healthy controls. Three further clusters represented transition zones between both DB levels (230–268) consisting of presymptomatic carriers with DB values increasing with decreasing distance from the cluster that indicated low DB and healthy age-matched controls.

**Conclusion:**

The proposed binary spin-bath MT method offers the potential to monitor DB and progression in HD. The method may augment qualitative MT techniques since it depicts tissue changes related to interactions between macromolecules and protons in disease specific brain regions that follow the neurodegenerative process.

## Introduction

Huntington’s disease (HD) is an autosomal dominant inherited disorder with abnormal movements, including chorea, dystonia, motor impersistence, and psychiatric and cognitive impairment (1, 2). The disorder is related to the elongation of a CAG triplet repeat in the huntingtin gene (HTT), a dynamic mutation, whereby the disease onset is inversely correlated with the number of repeats. Multiple aspects underlie cellular pathology in cells expressing the HTT gene with elongated triplet repeats (3–5). Among the numerous pathways disturbed in cells bearing HTT gene CAG triplet expansions are gene transcription and posttranslational protein handling, energy metabolism, intracellular protein trafficking and metabolism, dynamic axonal transport, or endocytic and vesicular trafficking changes. Complex interactions of these mechanisms lead to disturbed cellular function and to cell death. A specific degeneration is found in the striatum; however, numerous other regions get involved over time (6). Beside the loss of huntingtin function, its accumulation in cytoplasma and in nuclear inclusion bodies also plays a role (6). While methods to measure this accumulation have been readily established *in vitro*, there is a need to develop biomarkers for *in vivo* assessment.

Structural MRI has been recognized as a surrogate biomarker to assess volume changes of core structures in HD brains. New protocols now allow fully automated morphometry (7). Track-HD, a prospective study within the European HD Network (EHDN), included 366 premanifest individuals, patients with HD and controls. After a 1 year follow-up period, this study demonstrated that the rates of brain atrophy were higher in premanifest HD carriers and early HD patients compared to healthy controls even in persons far from predicted disease onset (8). Clinical impairment in premanifest and early HD was associated with specific gray matter loss in the striatal areas, the sensory and motor cortices for motor-dependent tasks, the visual cortex and cuneus, and widespread along the white matter, dependent on the underlying motor and neuropsychiatric testing.

Beyond T1-weighted imaging, advanced neuroimaging techniques as diffusion tensor imaging, T2*-weighted relaxation time mapping and MR spectroscopy (MRS) have been successfully applied to investigate microstructural alterations associated with HD severity (9). However, none of these methods allow assumptions about the amount of accumulated huntingtin with elongated polyglutamine track in the basal ganglia. Magnetization transfer (MT) imaging has an advantage in that it determines physical properties that depend on the presence of macromolecules not *directly* measurable by structural imaging techniques and MRS. MT transmits radio-frequency energy to protons bound to macromolecules within the tissue of interest, e.g., the gray matter of the human brain. Dependent on the macromolecule concentration, energy is transferred to free soluble protons via dipole–dipole interactions. In relation to the degree of coupling between bound and unbound protons, the free water pool becomes partially saturated and can be subsequently imaged using routine RF pulses and gradients. Model-based magnetization transfer (mMT) imaging is a refined MT method that goes beyond the clinically used MT ratio (MTR) and allows the investigation of the fundamental parameters that describe the basic MT interaction processes. In contrast to MTR, the MT process and the relaxation processes are well separated to avoid statistically misleading results (10). According to this separation of the fundamental MT and relaxation parameters, the interaction and coupling of the HD specific macromolecules with its environment can be fully described and related to the underlying pathological process.

In this study, we have investigated differences in the mMT parameters mentioned above in two cohorts of HD mutation carriers, one during clinically manifested disease and one during a preclinical stage without motor symptoms and cognitive decline in comparison to a group of healthy control persons. We hypothesized that changes in mMT parameters describing the restricted and free proton pool and the restricted-to-free proton pool size ratio would allow to identify different mMT profiles between HD carriers and patients.

## Materials and Methods

### Study Participants

Three cohorts of participants have been included in this prospective study, which has been approved by the ethics committee of the state of Bern. All participants gave informed consent or, in the case of a legal representative confirming, assent for clinical examination and for MMT studies. The cohort of affected gene carriers included male and female individuals, 18 or older, with clinically manifest HD and a known mutation including the exact number of CAG triplet repeats. At the time of examination, they were in a middle state of motor involvement with a total score higher than 30 according to the motor part of the unified Huntington’s Disease Scale (UHDRS) and a diagnostic confidence of motor manifestation of 5. The value of their independence scale was between 50 and 75%. Participants with premanifest HD were male and female HD mutation carrier, 18 or older. Their motor score in the UHDRS was lower than 4, and their value 100% on the independence scale. Control persons included men and women age 18 or older without neurological or psychiatric history and family history.

Exclusion criteria in all three groups included history or epileptic seizures or meningoencephalitis, known other brain lesions other than HD, severe claustrophobia, ongoing drug and/or alcohol abuse, and medication with amphetamines, methylphenidate, foscarnet, ganciclovir, ritonavir, cocaine, gamma-hydroxybutyrate, theophylline, chloroquine, mefloquine, imipenem, penicillin, ampicillin, cephalosporins, metronidazole, isoniazid, levofloxacin, cyclosporin, chlorambucil, vincristine, methotrexate, cytosine arabinoside, lithium, systemic antihistamines, and systemic sympathomimetics. Participants with magnetic (metallic) particles in the scull or brain, with a cardiac pacemaker or deep brain stimulators, were excluded, as well as patients that could not undergo MRI exam due to severe motion.

Gene carrier and HD participants were examined using the motor part of the UHDRS. The cognitive battery included the verbal fluency test with a three-letter fluency performed in each 1 min and a categorical fluency test (animals) for 1 min; the symbol-digit modality test; the Stroop test (color naming, reading, and interference); and the trail making tests A and B. The psychiatric evaluation was performed with the problem behavior assessment for HD, short form (11). The examination was done as part of the participation in the Registry protocol, version 3, of the EHDN. To compare the values of the groups, the disease burden score (DBS) was calculated according to the following formula: Age × (CAG repeat number in the larger allele − 35.5).

### mMT Imaging

**Figure 1:**
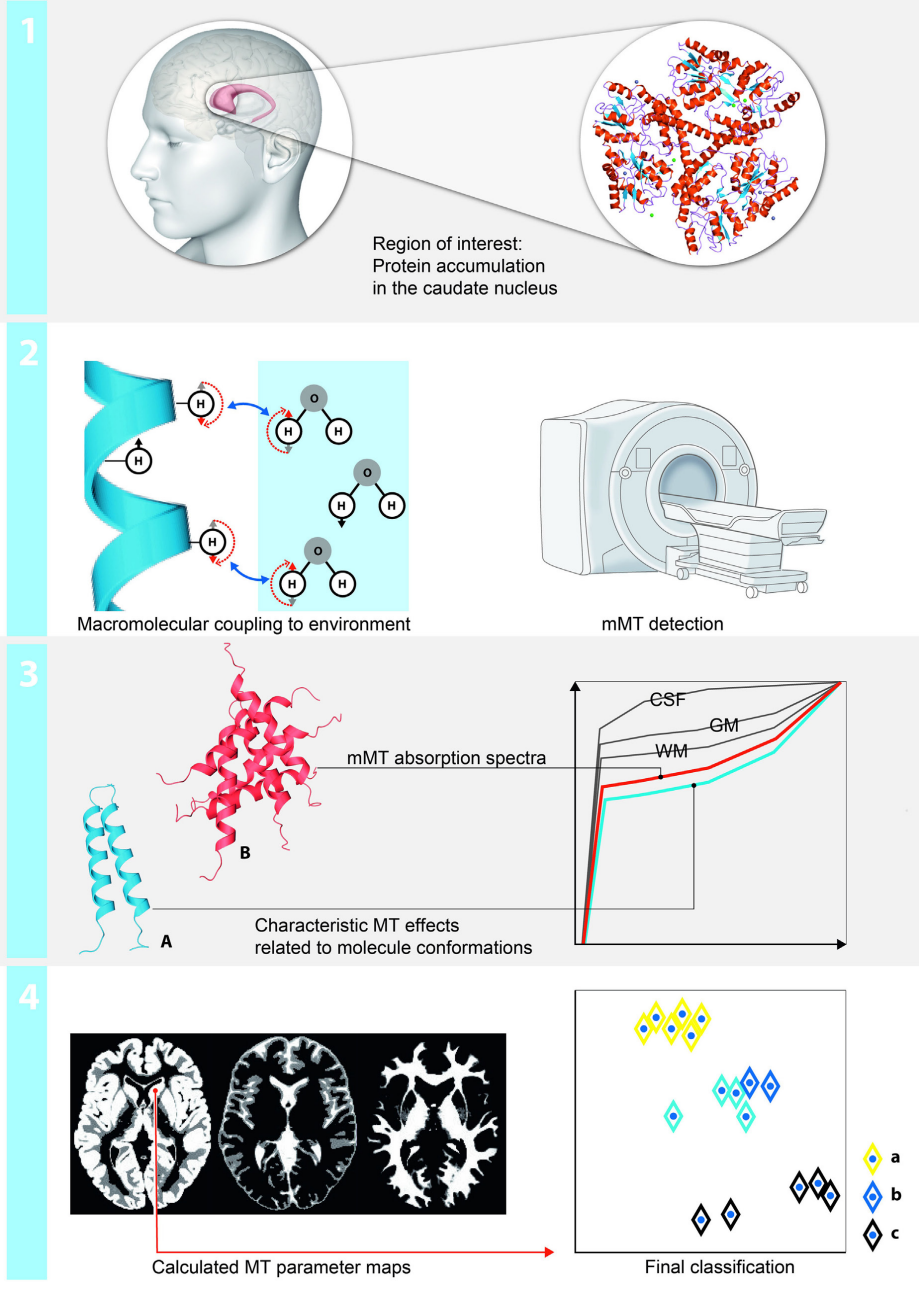
Schematic illustration of the region-based multiparametric model-based magnetization transfer (mMT) analysis: (1) mMT is sensitive to measures of macromolecular tissue accumulation. Changes in the restricted and free proton pool and the restricted-to-free proton pool size ratio in a region of interest analysis (caudate nucleus) are used to discriminate between presymptomatic and symptomatic stages of Huntington’s disease (HD). (2) The protons can be described as existing in two pools: as free or bound protons. The mMT model discriminates interstitial bulk (free) water deposits and the reservoir of restricted protons of macromolecules. The restricted-to-free proton pool size ratio quantifies the macromolecular over liquid content within an individual voxel and is hypothesized to be a proxy for the content of macromolecular storage in HD brains. (3) The experimental base for the quantification of the magnetization transfer effects is a repeated presaturation of the sample with Gaussian RF pulses irradiated at different off-resonant frequencies. Normalized mMT absorption spectra for each brain compartment (CSF, white matter, gray matter outlined in gray), the amount of abnormally accumulated large proteins (red) vs. physiological conditions (blue) enable groupwise stratifications of patients, pre-HD carriers and HC. (4) A fuzzy clustering algorithm with subsequent principal component analysis (PCA) for the projection on two dimensions is applied. The axes correspond to the dimensions of the projected data using the eigenvectors with the largest eigenvalues calculated by the PCA—of note, the other main components only contain noise and irrelevant information.

The protons in physiological systems as well as during pathological macromolecular accumulation can be described as existing in two pools: as free or bound protons. The mMT model discriminates interstitial bulk (free) water deposits associated with aging and atrophy (T2f) and the reservoir of restricted protons of macromolecules (T2r). The restricted-to-free proton pool size ratio (*F*) quantifies the macromolecular over liquid content within an individual voxel and is assumed to be a proxy for the content of macromolecular storage in HD brains. Spin-density free proton (SDf) is a marker for density of free protons within the tissue. We made use of a binary spin-bath MT model (12) to separate effects related to a direct saturation of the free pool and real MT effects and determining the fundamental physical parameters that characterize the shape of the MT spectra: the relative size of the restricted proton pool (*F*), the magnetization exchange rates between the free and the restricted pools (kf [free → restricted], kr [restricted → free]), the T2 relaxation time of the restricted pool (T2r), and the relaxation times T1 and T2f of the free pool, which were determined in separate experiments on the basis of the Bloch equations (13). The restricted-to-free proton pool size ratio (*F*) quantifies the macromolecular over free proton content within an individual voxel. The exchange rates (kf, kr) express the through-space transfer of magnetization between the reservoirs by magnetic dipole-dipole coupling and chemical exchange. The T2r relaxation time of the restricted pool can be interpreted as a marker for the type of the macromolecules and its coupling to the environment (13, 14). A schematic description of the method is provided in Figure 1.

### MR Sequence

The experiments were performed on a 3.0 T whole-body scanner (Siemens Magnetom Verio TIM, Siemens Erlangen, Germany) equipped with a 12 channel head coil. The acquisition time for the entire protocol (3D-T1, mMT) approximated 30 min.

The experimental base for the quantification of the MT effects is a repeated presaturation of the sample with Gaussian radio-frequency pulses irradiated at different off-resonant frequencies. The MTR was calculated by relating the MT images acquired at Δ*f* = 1.00 kHz to the data without presaturation (*M*0): MTR = 100 × [*M*0 − mol/L(Δ*f* = 1.0)]/*M*0. The MT-weighted images were acquired by using a set of seven gradient-echo FLASH sequences with Gaussian-modulated presaturation pulses at frequency offsets (Δ*f*) of 1.00, 2.00, 4.00, 8.00, 10.00, 12.00, and 16.00 kHz, according to the central 1H Larmor frequency [TR = 300 ms, TE = 4.18 ms, α_EXC_ = 20°, α_MT_ = 540° (Gaussian), section thickness = 4 mm (gap 0), 16 sections (interleaved), FOV = 256 mm, 128 × 128 matrix (data interpolated to 256 × 256)].

Structural MR imaging included a T1-weighted, sagittal-oriented 3D magnetization-prepared rapid acquisition of gradient-echo sequence (TR/TE/TI, 2,000/3.42/1,100 ms; matrix size 256 × 256; FOV, 256 mm × 256 mm; flip angle, 15°; slab, 160 mm) with a 1-mm^3^ isovoxel resolution.

### Data Extraction and Image Calculation

We focused the analysis on the mMT measures in the caudate nucleus (NC) as a single region-of-interest (ROI), since this brain area revealed well-replicated findings of huntingtin-related damage in prodromal-HD subjects (6). The non-linear fitting of the theoretic MT model-based signal to the measured MT signal intensity within each imaging voxel was accomplished by using a Levenberg–Marquardt algorithm, which provides the optimal parameter values for each voxel in a least-squares sense. Following this approach, complete new images were calculated that represent the different parameter values for each voxel (13, 14) for further ROI analysis. An expert neuroradiologist with 15 years of experience manually traced the NC on a reference data template in Montreal Neurological Institute space (SPM Anatomy toolbox, colin27T1^1^). The borders of the NC were identified on the T1w image as the gray matter adjacent to the lateral ventricle, from the caudate head at the rostral border of the internal capsule to its tail in close relationship to the amygdala following the caudate tracing guidelines by Westmoreland and Cretsinger.^2^ To project these region from a reference data template in Montreal Neurological Institute space (SPM Anatomy toolbox, colin27T1, see text footnote 1) into the individual brains, the SPM5 (Wellcome Department of Imaging Neuroscience, London, UK) warping algorithms and Jacobian matrices were used.

### Statistics

The classification was performed with a Gustafson–Kessel algorithm (15). The Gustafson–Kessel algorithm associates each cluster with both a point and a matrix, representing the cluster center and its covariance. This technique is capable of detecting hyperellipsoidal clusters of different sizes and orientations by adjusting the covariance matrix of data, thus overcoming the drawbacks of a conventional fuzzy-c-means algorithm. This choice is essential because it makes the classification more robust against outliers and noise.

After clustering in the high-dimensional space the essential classification is performed in a two-dimensional representation which is accomplished by a principle component analysis. The two main components with the largest eigenvalues were used for the final classification.

The program for the cluster analysis is an in-house-written software based on the Matlab (MathWorks, Natick, MA, USA) environment. The postprocessing procedure is fully automated and has been described in detail previously (13, 14).

## Results

### Participants

Twenty persons with increased numbers of CAG repeats on the larger *huntingtin* allele (39–49) were included. None of them underwent intubation. Ten symptomatic persons had 41–49 CAG repeats (5 women, age at examination 35–70 years old, mean 54), and 10 asymptomatic persons 39–47 CAG repeats (9 women, age at examination 23–60 years, mean 41). The symptomatic group was slightly older on average (*p* = 0.04), but their mean CAG repeat number did not differ (*p* > 0.05, paired *t*-test). The complete demographic characteristics and assessment of the clinical features are provided in Table 1. Ten individuals (five women, age at examination 23–64 years old, mean 48) without neurological or psychiatric disease were included as controls. An overview about the neuropsychological and behavioral tests results is provided in Table 2.

**Table 1:** Clinical data (clinically asymptomatic gene carriers and symptomatic HD patients).

Demographic data and clinical scores	Carriers, *n* = 10 (*f* = 9)		Manifest, *n* = 10 (*f* = 5)		Paired *t*-test
	Mean	SD	Mean	SD	
Age	40.6	9.1	54.2	12.7	0.04
CAG repeats	42.5	2.4	43.7	2.8	n.s.
Disease burden score	266.4	73.5	443.0	123.4	0.002
Total motor score	1.3	1.1	51.0	24.6	0.00001
Total functional score	5.7	0.3	12.9	3.4	0.0001

*UHDRS, Unified Huntington’s Disease Scale; m, male; f, female; HD, Huntington’s disease*.

**Table 2:** Neuropsychological tests (clinically asymptomatic gene carriers and symptomatic Huntington’s disease patients).

	Carriers (*n* = 10)		Manifest (*n* = 10)		
Word fluency (letters)	41.5	13.9	17.4	12.3	
**Stroop**					
	Naming	78.3	13.8	35.6	17.7
	Reading	86.4	18.1	40.7	20.6
	Interference	50.5	11.6	22.1	12.2
Symbol digit	48.8	6.8	18.2	11.0	
Word fluency (category)	23.2	4.1	10.1	5.5	
**Trail making**					
A	23.6	6.5	103.6	63.5	
B	70.4	22.6	207.6	50.5	
**Problem Behaviors Assessment (short)**						
	Depression	3.0	3.4	4.4	5.0
	Irritability aggression	1.6	2.0	2.7	2.6
	Psychosis	0.2	0.6	0.9	1.3
Apathy	0.2	0.6	4.1	3.8	
Executive function	0.0	0.0	6.0	4.8	

### Magnetization Transfer

**Figure 2:**
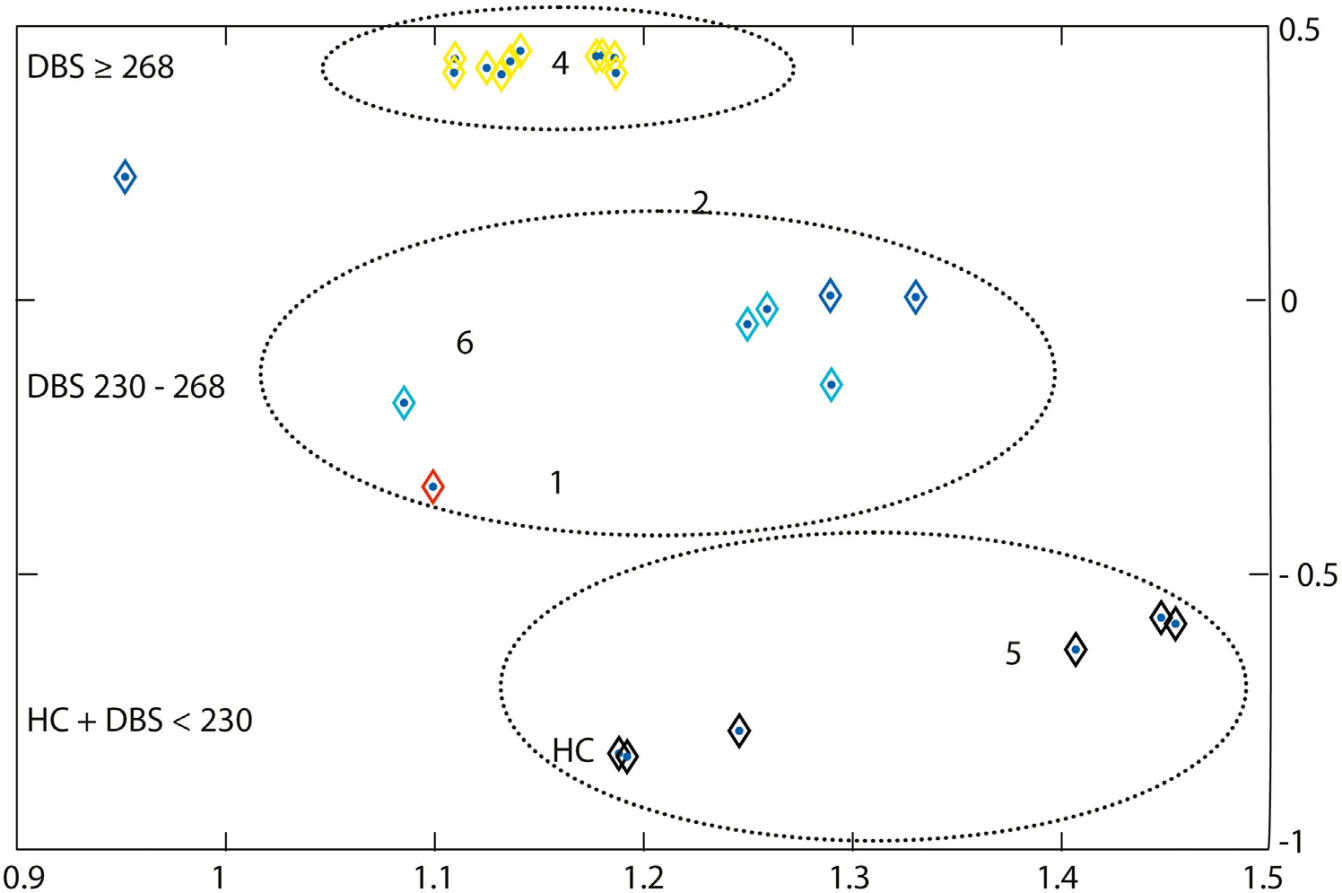
A region-of-interest cluster analysis of the nucleus caudatus was performed with fuzzy clustering to associate this subregion to a cluster representative for each group. The cluster analysis of the patients and controls was performed according to the MR parameter subset (T2f, SDf, kf, kr, T2r), given a maximal number of six classes. Each subgroup (symptomatic, presymptomatic, and control) consists of 10 persons. Cluster 4 represents subjects with a disease burden score (DBS) of ≥268 (yellow), it includes all symptomatic Huntington’s disease patients and one non-symptomatic gene carrier. Cluster 5 represents the non-symptomatic gene carrier with DBS <230 and controls (black). Clusters 1, 2, and 6 (red, light and dark blue) essentially represent transition zones consisting of presymptomatic patients with DBS values increasing with decreasing distance from cluster 4. Cluster 3 is empty. The axes correspond to the largest eigenvalues found by the principal component analysis.

**Figure 3:**
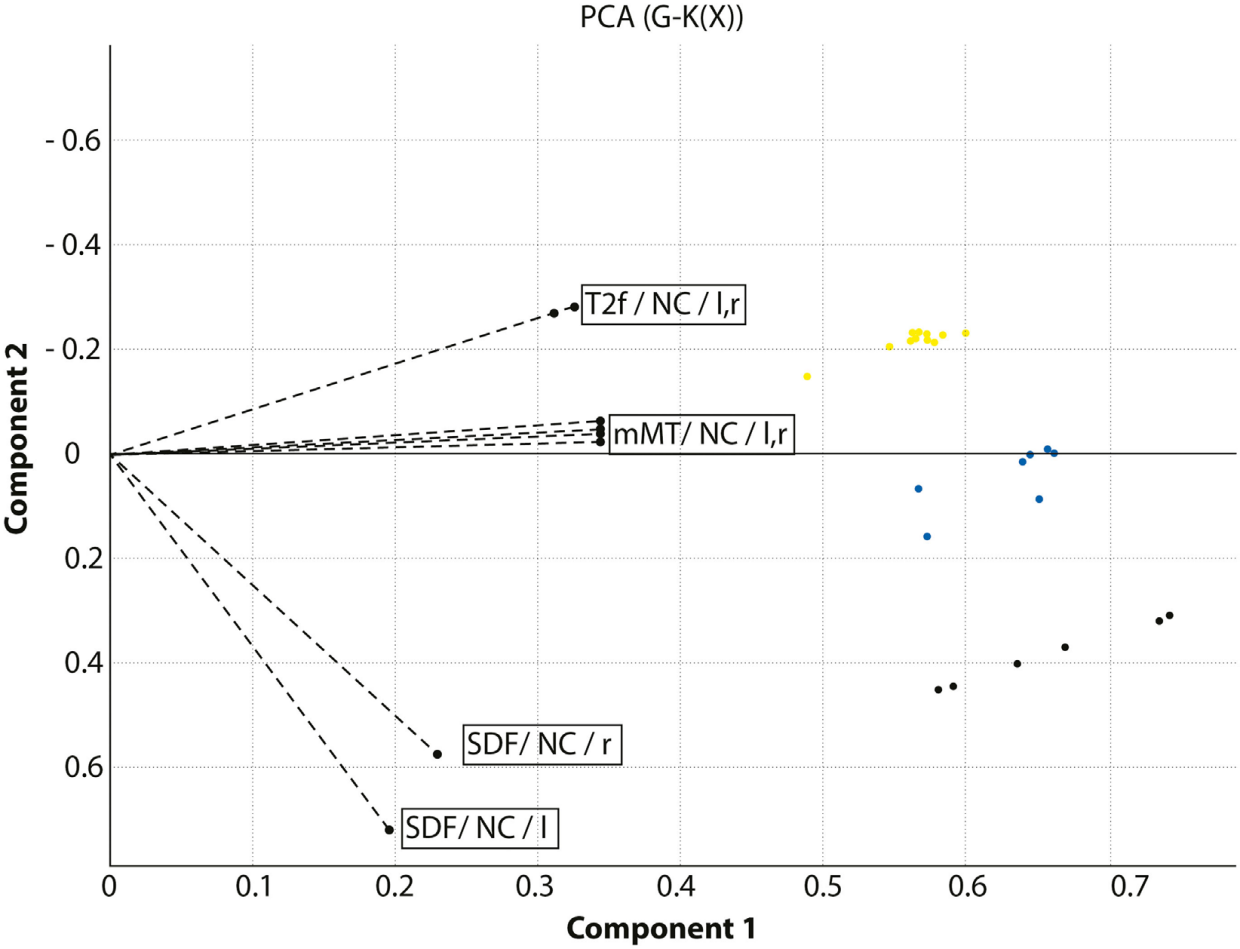
Combined presentation of the factor loadings and the classification of the cohort under investigation. The axes indicate the main components of the principal component analysis. The dotted lines indicate the relative contribution of the model-based magnetization transfer (mMT) parameters T2f, SDf, kf, kr, and T2r extracted from the caudate nucleus (NC) in the left and right hemisphere to the segregation between HC, Huntington’s disease (HD) carriers and manifest HD patients. HD patients are displayed in yellow, HD carriers in blue, and healthy controls in black.

In the ROI cluster analysis of the NC, fuzzy clustering was performed to associate this subregion to a cluster representative for each group. The cluster analysis of the patients and controls was performed according to the MR parameter subset (T2f, SDf, kf, kr, T2r), given a maximal number of six classes. There was a clear separation into two main clusters: the first cluster encompassed HD mutation carriers with a disease burden (DB) exceeding 268 (*n* = 11). The other important cluster contained the normal controls and cases with low DB. In between were cases with medium DBS, and this encompassed three clusters (Figure 2). We computed the factor loading—the relative contributions of the individual mMT parameter to the main components (first and second principal component). The first two components explain 94.4% of the variance. The first component that is dominated by the mMT parameters explains 84% of the variance. From Figure 3, it can be depicted that the individual contributions of each mMT parameter is essential for the segregation and that the classification is mainly driven by the characteristic macromolecular interactions and not simply by atrophy. We further calculated the individual gray matter/total intracranial volume ratios for patients (0.42 ± 03), carriers (0.48 ± 03), and HC (0.48 ± 03). While there was as significant difference between patients and carriers/HC (*p* < 0.001), we identified no such differences between carriers and healthy controls (*p* = 0.7, one-way ANOVA).

## Discussion

In this pilot study, we investigated the potential of a region-based mMT analysis of the NC to segregate symptomatic HD gene carriers from healthy controls and non-affected HD carriers. Automated fuzzy clustering of the patients encompassing several mMT parameters (T2f, SDf, kf, kr, T2r) classified patients with a high DBS >268 (including one asymptomatic patient with high DBS) from individuals with intermediate DBS between 230 and 268 and from those with a low DBS (<230) and normal elderly controls with a sensitivity of 97%. Asymptomatic HD carriers were segregated from HC by mMT along a “transition zone” that corresponded to increasing DB values. The morphometric analysis of GM/TIV volume ratios did—in contrast to the mMT parameters—not discriminate between carriers and HC. These finding suggest a relationship between DBS and load of macromolecules in the NC. As an exception, one asymptomatic gene carrier with a high DBS was categorized within the cluster of HD patients. This “erroneous” clustering emphasizes a drawback of clinical scoring systems in defining a clear cut symptom onset beyond a consideration solely based on progressing motor symptoms. In HD, similar to other neurodegenerative disorders, cognitive and/or behavioral decline may appear subtle and unrecognized far prior to motor ones, and thus a revision of the diagnostic criteria has been suggested (16).

Volumetric structural image analysis by MRI is the accepted standard procedure to quantify atrophy in HD. Decreased caudate and putamen volume distinguishes subjects with HD normal controls and has been demonstrated to track disease progression. Biomarker studies as TRACK-HD and PREDICT-HD revealed promising results in the assessment of disease progression in premanifest and early HD (17, 18). A volume reduction predominantly in the putamen to intracranial volume ratio, further in the NC, hippocampus, globus pallidus, nucleus accumbens and thalamus, and lobar gray matter, but not in the lobar white matter, and an increase in volume of cerebral fluid was associated with disease progression when using an automated segmentation procedure. However, a potential limitation of volumetric measures in drug studies is that potential effects may not necessarily decrease with brain atrophy in the presence of a meaningful clinical benefit. Quantitative mMT imaging in this sense, offers the opportunity to directly assess macromolecular tissue compositions presumably reflecting microstructural damage related to HD pathology independently from brain atrophy. Notably, we incorporated solely the NC into our analysis, since it reflects the candidate area for earliest microstructural changes. One early phenomenon described in several models is the formation of inclusion bodies made of abnormal HTT with elongated polyglutamate track in nuclei and dystrophic neuritis (19). The mechanisms underlying the formation of these inclusion are complex, the elongation of the protein itself may lead to a disturbed intracellular clearance with accumulation, but decreased apoptotic signaling, increased cytosolic calcium concentration, and relative NMDA receptor affinity among other mechanisms, may play a role as well (20). The burden of these inclusions correlates with the number of CAG triplet repeats (21). The formation of HTT inclusion bodies is accompanied by changes in the cytosolic macroprotein conformations. Accumulations of various proteins, including heat-shock protein chaperones, ubiquitin, and other components of the proteasome system (21) and numerous protein–protein interactions perturbed by elongated HTT (22) may affect the interactions between free and restricted protons and thus reflect physical properties in the presence of solid structural components detectable by mMT.

Several studies have addressed MT imaging, yet focusing on the MTR. A reduction of the MTR peak height in gray and white matter in manifest HD compared to healthy controls (23) has been reported, but no differences between presymptomatic gene carriers and controls. These studies concluded that there is no role for MTR as a biomarker in presymptomatic HD (24). The shortcomings of these studies are their strict focus on MTR: MTR is influenced by T1 effects and T2-relaxometry and does not differentiate between effects related to the fractional deposit of macromolecules and those related to the coupling of restricted protons. MT imaging has been consistently applied as an overall measure to various other neurodegenerative disorders, e.g., Alzheimer disease (25), Parkinson disease (26, 27), and progressive supranuclear palsy (28), however, MTR lacks the specific properties to identify the constituents of tissue that are essential to identify all tissue components that dominate the MT exchange process in GM separately. Very recently, a similar method has been applied to investigate HD-related myelin breakdown in WM (29). Quantified MT parameters that reflect the relative density of the macromolecular pool were highly sensitive to microstructural changes related to the myelin content of WM in HD patients. Quantified mMT is straightforward also in GM, since it is targeted against disease-related macromolecule deposits instead of volumes in the cortex or white matter which tend to be affected later during the disease process.

Our pilot study provides first evidence that this method may be also of potential value in the classification of gene carriers as a potential marker of disease progression, unrelated to global atrophy. MT parameters that reflect an increased load of interstitial water (T2f) and that are indicators of aging and atrophy, did not result in a clustering modifications in a previous study on pre-AD subjects (14), indicating that structural changes are minor during early preclinical stages. At the current stage the complete set of mMT parameters provided the most accurate classification with respect to the DBS, as can be seen from the distribution of the factor loadings displayed in Figure 3. This observation can be explained by the fact that the complexity of the interaction of macromolecules with its immediate environment cannot be adequately described by a single oversimplified parameter such as the MTR whereas the MT model parameters account for the associated complex exchange and coupling phenomena.

This study has limitations. The number of patients was small, and the investigations were focused on a PCA supervising an established fuzzy clustering algorithm to segregate controls, gene carriers, and symptomatic individuals. To investigate the relationship strength between the clustering with clinical phenotype assessment, larger longitudinal studies are necessary to characterize macromolecule conformations, sensitivity and specificity of the individual mMT parameters. Other multivariate statistical methods, as, e.g., discriminant function analysis may be alternatively employed to test the prediction accuracy of mMT and subsequent disease monitoring from prodromal stages into HD *in vivo*.

Further *in vitro* experiments of the concentration-related interactions of macromolecules are also mandatory, using synthetic biological multi-compartment matrices that contain various macromolecules (huntingtin, ubiquitin, and tau-protein) and different synthetic environments.

Multiparametric MRI protocols that record a compound of several biomarkers reflecting atrophy, protein load, and metabolic processes within a single MR exam have meanwhile become feasible with reasonable overall acquisition times of 30–40 min that render mMT applicable as a complement for HD imaging protocols in future clinical trials.

## Ethics Statement

Prospective study has been approved by the ethics committee of the state of Bern. All participants gave informed consent or, in the case of a legal representative confirming, assent for clinical examination and for MMT studies.

## Author Contributions

RW concepted the study, contributed to data acquisition and analysis, and wrote the paper. J-MB contributed to data acquisition, clinical and statistical data analysis, and writing of the manuscript. CK concepted the study, performed the MR data analysis, and contributed to the writing of the manuscript.

## Conflict of Interest Statement

The authors declare that the research was conducted in the absence of any commercial or financial relationships that could be construed as a potential conflict of interest.

## Footnotes

^1^https://www.fz-juelich.de/SharedDocs/Downloads/INM/INM-1/DE/Toolbox/Toolbox_22c.html?nn=563092.

^2^https://www.icts.uiowa.edu.

## Abbreviations

DB, disease burden; HD, Huntington’s disease; HTT, huntingtin gene; mMT, model-based magnetization transfer; MT, magnetization transfer; MTR, MT ratio; PBA, Problem Behaviors Assessment; UHDRS, unified Huntington’s Disease Scale.
